# Protein Function Prediction using Text-based Features extracted from the Biomedical Literature: The CAFA Challenge

**DOI:** 10.1186/1471-2105-14-S3-S14

**Published:** 2013-02-28

**Authors:** Andrew Wong, Hagit Shatkay

**Affiliations:** 1 Computational Biology and Machine Learning Lab, School of Computing, Queen's University, Kingston, ON, K7L 3N6, Canada; 2 Dept. of Computer and Information Sciences, University of Delaware, Newark, DE, 19716, US; 3 Delaware Biotechnology Institute, University of Delaware, Newark, DE, 19711, US

## Abstract

**Background:**

Advances in sequencing technology over the past decade have resulted in an abundance of sequenced proteins whose function is yet unknown. As such, computational systems that can automatically predict and annotate protein function are in demand. Most computational systems use features derived from protein sequence or protein structure to predict function. In an earlier work, we demonstrated the utility of biomedical literature as a source of text features for predicting protein subcellular location. We have also shown that the combination of text-based and sequence-based prediction improves the performance of location predictors. Following up on this work, for the Critical Assessment of Function Annotations (CAFA) Challenge, we developed a text-based system that aims to predict *molecular function *and *biological process *(using Gene Ontology terms) for unannotated proteins. In this paper, we present the preliminary work and evaluation that we performed for our system, as part of the CAFA challenge.

**Results:**

We have developed a preliminary system that represents proteins using text-based features and predicts protein function using a k-nearest neighbour classifier (*Text-KNN*). We selected text features for our classifier by extracting key terms from biomedical abstracts based on their statistical properties. The system was trained and tested using 5-fold cross-validation over a dataset of 36,536 proteins. System performance was measured using the standard measures of precision, recall, F-measure and overall accuracy. The performance of our system was compared to two baseline classifiers: one that assigns function based solely on the prior distribution of protein function (*Base-Prior*) and one that assigns function based on sequence similarity (*Base-Seq*). The overall prediction accuracy of *Text-KNN, Base-Prior*, and *Base-Seq *for *molecular function *classes are 62%, 43%, and 58% while the overall accuracy for *biological process *classes are 17%, 11%, and 28% respectively. Results obtained as part of the CAFA evaluation itself on the CAFA dataset are reported as well.

**Conclusions:**

Our evaluation shows that the text-based classifier consistently outperforms the baseline classifier that is based on prior distribution, and typically has comparable performance to the baseline classifier that uses sequence similarity. Moreover, the results suggest that combining text features with other types of features can potentially lead to improved prediction performance. The preliminary results also suggest that while our text-based classifier can be used to predict both *molecular function *and *biological process *in which a protein is involved, the classifier performs significantly better for predicting *molecular function *than for predicting *biological process*. A similar trend was observed for other classifiers participating in the CAFA challenge.

## Introduction

Proteins play a fundamental role in all living organisms and are involved in a variety of molecular functions and biological processes. Thus, characterizing the function of proteins is an important goal in proteomic research. Due to high-throughput sequencing technologies, the number of proteins whose sequence is available but whose function is still unknown is growing rapidly. Unfortunately, experimental procedures for studying protein function are still costly and time consuming. Therefore, computational prediction systems are actively being developed and used to help deduce protein function. The Critical Assessment of Function Annotations (CAFA) Challenge [1] aimed to evaluate a variety of systems performing a shared task of predicting protein function, where the function categories were based on the *molecular function *and *biological process *components of the Gene Ontology (GO).

Traditionally, computational prediction methods use features that are derived from protein sequence, protein structure or protein interaction networks to predict function [2-5]. For example, GOtcha [6], OntoBLAST [7], and BLAST2GO [8] assign protein function based on sequence similarity; PHUNCTIONER [9] and ConFunc [10] predict function based on similarity between protein structures; systems that use protein interaction networks to deduce function include GeneMANIA [11] and the prediction system developed by Chua *et al. *[12].

In contrast, we propose a system that uses text from the biomedical literature as a source of features to predict function; these features are used to represent proteins and to assign functional annotation. We have submitted our preliminary results to the CAFA challenge, and present here the fundamentals of our text-based prediction system.

There are two main ways to use text data in the context of inferring protein function: *Information Extraction *and *Classification*. The system presented here belongs to the latter category, namely, classification. Systems that use the *information extraction *approach aim to identify and extract phrases or terms from text sources that directly describe the function of a protein. That is, rather than *predict *protein function, such systems aim to *find what is already known *and reported in the literature about the function or the process in which a protein is involved. In contrast, a classification system uses features that are derived from text sources in order to represent any protein - regardless of whether its function is reported in the text or not. The system uses the text-based representation of proteins whose function is already known to train machine-learning classifiers that can then assign function labels to yet-unannotated proteins (where the latter are also represented using text-based features).

One of the earliest information extraction systems, AbXtract [13], identified sentences that discuss the function of proteins and ranked the relevance of the sentences according to the statistical significance of the words that are present. Since then, different information extraction systems have used a variety of strategies to retrieve passages of text that discusses protein function. For example, there are systems that use pattern matching and sentence structure analysis to retrieve sentences that contains both a protein name and a Gene Ontology (GO) term, from biomedical abstracts [14,15]. There are also systems that extract keywords from literature or databases, and associate the keywords with GO categories by using a dictionary-based approach [16] or by using clustering techniques [17]. Other information extraction systems are used within the biomedical domain to discover biological knowledge (see for instance, surveys by Jensen *et al. *[18] and by Cohen *et al. *[19]).

As for systems that perform classification based on text features, several systems have focused on annotating the function of yeast proteins. However, these systems are limited in scope as they only assign GO categories from the *biological process *ontology and these systems are only evaluated on a small number of proteins. For example, Raychaudhuri *et al. *[20] built a document classifier that assigns *biological process *GO categories to abstracts. The GO categories that are assigned to each abstract are then transferred to the proteins that are referenced in the abstract. Their system used 21 *biological process *terms from GO as function categories and was tested only on 1,188 *yeast proteins*, The text-based classification system by Nenadic *et al. *[21] used support vector machines to classify 2,975 yeast proteins into one of eleven *biological process *terms found in the higher level of the GO hierarchy. (By "higher level" of the GO hierarchy, we refer to the more general concepts of the hierarchy, as opposed to the most specific concepts that occur toward the leaves.) In their work, the proteins are represented by the words that are found within associated abstracts for each protein. The main goal of their work was to study the impact of various levels of text pre-processing on classification performance. They concluded that text pre-processing can significantly reduce the training time of the classifier without decreasing performance.

There are also text-based classifiers that classify biomedical abstracts, rather than proteins, into functional categories. The system by Theodosiou *et al. *[22] classified abstracts into 12 function categories (out of the 21 GO codes used in [20]) using *linear discriminant analysis*. Similarly, Pan *et al. *[23] used *linear discriminant analysis *to identify abstracts that describe associations between transcription factor proteins and GO categories.

Another text-based prediction system that is strongly related to the one presented here is EpiLoc [24]. EpiLoc is a text-based classification system that is used to predict protein subcellular localization. In that work, protein information was collected from the curated database UniProtKB/Swiss-Prot [25], which was used to train and test the classifier. In order to represent the proteins using text, the abstracts mentioned for each protein in the UniProtKB/Swiss-Prot database were retrieved from PubMed; Text features were then selected from the associated abstracts based on the *Z-Score *statistical test. While the focus of EpiLoc is protein subcellular localization as opposed to function, the system was compared to other state-of-the-art systems that use other types of features and showed that the text-based system offered competitive performance.

Toward the CAFA challenge, we aimed to develop a text-based classifier for representing and classifying proteins by their molecular function and biological process. We based our system on the framework introduced in EpiLoc, while modifying it to perform function prediction as opposed to protein subcellular localization. The trained system was applied through the CAFA challenge to proteins from both the eukaryotic and prokaryotic tracks of the CAFA evaluation dataset. We have trained and tested our system using 5-fold cross validation on a dataset of 36,536 proteins extracted from the curated protein database UniProtKB/Swiss-Prot. The proteins that we included in our dataset had at least one GO functional annotation with an experimental evidence code, and one associated abstract from which text features could be obtained.

The results from this evaluation, along with the fundamental aspects of this preliminary system and results from the CAFA challenge itself are presented in this paper. Notably, the system we submitted solely uses text-based features, and in-and-of itself is not expected to outperform other classifiers. The ultimate goal is to integrate the text-based system with a other types of function predictors, as our experience with location prediction [26] suggests that such an integration is likely to prove beneficial.

## Methods

To train our classifier, we compiled a dataset of proteins for which a reliable function or process annotation was assigned according to UniProtKB/Swiss-Prot, as described below. For each protein we also retrieved the PubMed abstracts that are referenced from its respective UniProtKB entry, as a source of text features. From the associated abstracts, we extracted terms that are characteristic for each functional category - that is, terms whose occurrence probability is statistically significantly different in abstracts of proteins in one functional class than in all other classes. We used these terms as text features to represent proteins. The dataset of proteins was then used to train and test our text-based classifier using 5-fold cross validation. Further details are provided below.

### The protein and the text datasets

Our dataset of proteins was extracted from the UniProtKB/Swiss-Prot database. We included proteins that are annotated with at least one GO category from either the *biological process *or *molecular function *ontology, and whose entry in the database includes at least one reference to a PubMed abstract. However, since we aimed to identify features that can characterize well each GO category corresponding to function or to process, we excluded from the dataset proteins that were annotated with three or more GO categories from the second level of the GO hierarchy. Furthermore, to ensure that the dataset consists of proteins whose annotation is of high certainty, we checked the *evidence code *associated with each GO annotation, and did not include in the dataset any annotations that were generated through computational methods. The evidence codes for annotations that are included in the dataset are shown in the left column of Table 1, while the evidence codes for which annotations were excluded are listed in the right column,

**Table 1:** List of evidence codes

Included evidence codes		Excluded evidence codes	
EXP	Inferred from Experiment	ISS	Inferred from Sequence/Structural Similarity

IDA	Inferred from Direct Assay	ISO	Inferred from Sequence Orthology

IPI	Inferred from Physical Interaction	ISA	Inferred from Sequence Alignment

IMP	Inferred from Mutant Phenotype	ISM	Inferred from Sequence Model

IGI	Inferred from Genetic Interaction	IGC	Inferred from Genomic Context

IEP	Inferred from Expression Pattern	RCA	Reviewed Computational Analysis

IC	Inferred by Curator	IEA	Inferred from Electronic Annotation

TAS	Traceable Author Statement	NAS	Non-traceable Author Statement

The table shows which GO evidence codes were included in our dataset and which evidence codes were excluded.

The final dataset thus consisted of a total of 36,536 proteins of which 21,764 proteins were annotated by GO categories from the *biological process *sub-ontology, and 22,309 proteins annotated with *molecular function *GO categories. This dataset was used for training and testing the system using 5-fold cross validation.

In order to represent proteins using text-features, we first linked each protein to the set of PubMed abstracts that are associated with it, based on UniProtKB/Swiss-Prot. To do so, we identified for each protein all the PubMed identifiers (PMIDs) that were listed in its UniProtKB/Swiss-Prot entry, and retrieved the corresponding abstracts from PubMed. Notably, our primary goal is to represent proteins using terms that are highly predictive of their potential function. Since a single abstract may be associated with multiple protein entries in UniProtKB/Swiss-Prot, which may have different functions, we excluded abstracts that are associated with more than three proteins that have different functions. Altogether, our text corpus consists of 68,337 abstracts for all the proteins in the dataset.

### The function-classes used

As per the CAFA Challenge requirements, our function classes consist of GO categories from both the *biological process *and *molecular function *ontologies. There are about 20,000 distinct GO categories in the *biological process *sub-ontology and about 9,000 categories in the *molecular function *sub-ontology. Ideally, each of the individual GO categories should correspond to a separate function class. However, this simple view cannot be directly realized. The reason being that many of the specific GO categories denoting function or process do not have a sufficient number of proteins (or associated PubMed abstracts) to be used for training a classifier. For example, the GO category '*platelet activating factor metabolism*' has only a single protein associated with it. When the number of training proteins for a function class is low we cannot extract text features that have a statistically significant association with the class.

For all these reasons, we do not use all the GO categories as function classes, but rather use a coarser class granularity. We thus use as function classes only GO categories that are at the second level of the GO hierarchy (one level away from the root node), merging together all the descendant GO categories below each node all the way down to the leaf-nodes. However, even at this level, some of the GO categories still do not have a sufficient number of proteins for training and testing a classifier. We therefore removed from the list of function-classes GO categories that are associated with fewer than 15 proteins (removing from the set both the GO categories and the associated proteins). This process resulted in a total of 35 function classes of which 24 are *biological process *GO categories and 10 are *molecular function *GO categories, all in the second level of the GO hierarchy. The resulting set of GO categories, which are used as function classes by our system, is shown in Table 2.

**Table 2:** The GO categories that are used as function classes in this work

Molecular Function		Biological Process	
**GO ID (#)**	**GO Category**	**GO ID (#)**	**GO Category**

0005488	Binding	0065007	biological regulation

0003824	catalytic activity	0032502	developmental process

0030528	transcription regulator activity	0009987	cellular process

0005215	transporter activity	0050896	response to stimulus

0060089	molecular transducer activity	0008152	metabolic process

0030234	enzyme regulator activity	0051234	establishment of localization

0005198	structural molecular activity	0016043	cellular component organization

0016247	channel regulator activity	0023052	Signalling

0009055	electron carrier activity	0032501	Multi-cellular organismal process

0045182	translation regulator activity	0022414	reproductive process

		0051704	multi-organism process
		
		0040011	Locomotion
		
		0040007	Growth
		
		0051179	Localization
		
		0022610	biological adhesion
		
		0008283	cell proliferation
		
		0000003	Reproduction
		
		0002376	immune system process
		
		0016265	Death
		
		0071554	cell wall organization or biogenesis
		
		0048511	rhythmic process
		
		0023046	signalling process
		
		0044085	cellular component biogenesis
		
		0043473	Pigmentation

### Text feature selection

The purpose of feature selection is to identify terms that can be used to characterize and to distinguish among proteins from different function classes. Such terms are the ones that are associated with a class with high statistical significance, as explained below. The selected terms are used as text features to represent proteins and to train our classifier. For our feature selection step, we adopt the approach introduced by Brady and Shatkay [24,26] in their work on predicting protein location. The main steps are described next.

We first pre-processed all the abstracts, extracting all individual words (unigrams) and all pairs of consecutive words (bigrams). We then applied the Porter Stemmer [27] to all the words, thus removing suffixes and reducing the words to their root form. Last, we reduced the number of terms by removing stop words such as '*or*', '*and*', or '*this*', as well as common words that appear in more than 70% of the abstracts. We also removed words that are rare and specific, which appear in fewer than three abstracts.

After pre-processing the abstracts to obtain the set of candidate terms, we use the *Z-Score *statistical test to find *characteristic terms *[24]. To avoid any use of information about the test data in this feature selection process, the Z-score-based feature selection uses only the training set within each 5-fold cross validation run [28]. That is, the *characteristic terms *are selected only from the four parts of the dataset that are used for training within each iteration of the cross validation. The test set (the fifth part of the dataset) is then represented based on the features selected from the rest of the dataset.

A term is considered to be *characteristic *with respect to a function *f*, if its probability to appear in abstracts associated with proteins whose function is *f *is statistically-significantly different from its probability to appear in abstracts associated with proteins of all other functions. For each term *t*, we compute the *Z-score *to measure the statistical significance of the difference in term occurrence probability across function classes. For a term *t*, and functions *f *and *f'*, the *Z-score *is defined as:

$$Z_{f,f'}^{t}=\frac{\text{Pr(}t\text{|}f\text{)-Pr(}t|f'\text{)}}{\sqrt{\dot{\text{P}}\cdot \text{(1-}\dot{\text{P}}\text{)}\cdot (\frac{1}{\left|D_{f}\right|}+\frac{1}{\left|D_{f'}\right|})}},\;where\;\dot{\text{P}}=\frac{\left|D_{f}\right|\cdot \mathrm{Pr}(t|f)\left|D_{f'}\right|\cdot \mathrm{Pr}(t|f')}{\left|D_{f'}\right|+\left|D_{f'}\right|}$$

and Pr(*t | l*) denotes the conditional probability of term *t *to appear in abstracts that are associated with proteins whose function is *l*. For any function *l*, the conditional probability Pr(*t | l*) is estimated using a maximum likelihood estimate, by dividing the number of abstracts that contain the term *t *and are associated with proteins whose function is *l*, by the total number of abstracts associated with proteins whose function is *l*. Formally, this conditional probability is defined as:

$$\text{Pr}\left(t|l\right)\approx \frac{\left|d\in D_{l}\;s.t.\;t\in d\right|}{\left|D_{l}\right|}$$

where *d *denotes an individual abstract and *D_l _*denotes the set of abstracts that are associated with proteins whose function is *l*.

If the absolute value of the Z-score for a term *t *and a function *f *is higher than a predetermined threshold with respect to all other functions *f'*, the term *t *is selected as a *characteristic term *for function *f*.

In our system, we use the union of *all the characteristic terms *over all function classes as the set of text features for representing proteins. For the *molecular function *classes, a total of 521 *characteristic terms *were selected and for the *biological process *classes, a total of 831 *characteristic terms *were used.

### Representing proteins using feature vectors

We represent proteins using the '*bag of words*' approach [29], as briefly explained below. The selected set of characteristic terms, denoted as *T_N_*, is used to represent each individual protein *p *as a *v*ector of | *T_N _*| term weights. (We thus use 521-dimensional vectors in the case of the molecular function classifier and 831-dimensional vectors for the biological process classifier). Each term weight, $w_{t_{i}}$, represents the significance of the characteristic term *t_i_*, within the set of abstracts associated with protein *p *(the set of abstracts is denoted as *D_p_*). The term weight is calculated as the ratio between the number of times term *t_i _*appears within *D_p _*and the total number of term occurrences of all distinguishing terms, *t_j, _*from the set *T_N _in D_p_*:

$$w_{t_{i}}^{p}=\frac{\#\;of\;times\;t_{i}\;appears\;in\;D_{p}}{\Sigma_{t_{j}\in T_{N}}\left(\#of\;times\;t_{j}\;appears\;in\;D_{p}\right)}.$$

In the evaluation set that was given by CAFA, 65 of the 596 proteins had no associated abstracts. For such "*textless*" proteins the text features of homologous proteins are used (as was done before in EpiLoc [24]). We use BLAST to compare the sequences of *textless *proteins to the sequences of proteins that have associated abstracts. We rank the BLAST results by their e-values and choose the three proteins with the lowest e-values as homologs. We then assign a weighted combination of the three feature vectors to the *textless *protein. To account for the degree of homology between the *textless *protein and its homologs, we multiply the term weights of each feature vector by the percentage of matched amino acids that are identical (*percent identity*). We then divide the term weights of each feature vector by three and sum the resulting weighted feature vectors together to obtain a representation for the *textless *protein.

### Training the classifier

As previously mentioned, we use a k-nearest neighbour (kNN) classifier to classify un-annotated proteins. The *cosine coefficient *between feature vectors is calculated and used as a similarity measure to find the nearest neighbours. It is defined as the cosine of the angle between two vectors:

$$\text{cos}\left(p,q\right)=\frac{\sum_{i=1}^{n}p_{i}\times q_{i}}{\sqrt{\sum_{i=1}^{n}{\left(p_{i}\right)}^{2}}\times \sqrt{\sum_{i=1}^{n}{\left(q_{i}\right)}^{2}}},$$

where *p *and *q *are the two *n-*dimensional vectors.

The 10 nearest neighbours are then used to classify unannotated proteins. It is common practice [30] to assign an un-labeled item to the class that is shared by the majority of its nearest neighbours. However, since a protein can have multiple functions, we modified the kNN classifier to assign all functions that are shared by three or more of the 10 nearest neighbour to an un-annotated protein.

We also return a confidence score for each prediction made by the classifier. For an un-annotated protein, *p*, and a predicted function class, *f*, the confidence score *C_f _(p*) is calculated as:

$$\begin{array}{cc} C_{f}\left(p\right)=\frac{\sum_{i=1}^{\left|N^{f}\right|}\text{cos}\;\left(N_{i}^{f},p\right)}{\left|N^{f}\right|},\; & \;3\leq \left|N^{f}\right|\leq 10 \end{array}$$

where, out of the 10 nearest neighbours of protein *p*, $\left|N^{f}\right|$ is the number of nearest neighbors with function *f*, and $\text{cos}\;\left(N_{i}^{f},p\right)$is the *cosine coefficient *between *p *and $N_{i}^{f}$, its *i*'th nearest neighbor with function *f*. The average of the *cosine coefficient *values calculated between protein *p *and each of its neighbors $N_{i}^{f}$ is then used as the confidence score.

### Evaluating the classifier

To evaluate the performance of our classifier (*Text-KNN*), we perform stratified 5-fold cross-validation using the dataset of 36,536 proteins described above. The dataset is partitioned at random into 5 disjoint subsets where each subset retains the same distribution of class instances as in the original dataset. The classifier is evaluated five times, where in each run a different subset of the data is used for testing while the remaining four subsets are used for training the classifier. To ensure the robustness of our results, we perform five complete sets of 5-fold cross-validation (totaling 25 runs altogether).

We compared our system performance to that of two baseline classifiers. The first baseline denoted *Base-Prior *assigns a class to a protein based on the prior distribution of function classes in the training set. For instance, if in the dataset 60% of the proteins belong to the function class '*binding*' and 40% of the proteins belong to the class '*catalytic activity*', assigning a class label to a protein *p *is done by Monte-Carlo sampling from a label distribution with a 60% chance of obtaining the label '*binding*' and 40% chance of obtaining the label '*catalytic activity*', and assigning the sampled label to *p*. The protein *p *will thus be assigned the label '*binding*' by the classifier with a probability of 0.6 and the label '*catalytic activity*' with probability of 0.4.

The second baseline classifier, denoted *Base-Seq*, assigns function classes based on sequence similarity. Given a protein *p*, the classifier uses BLAST (with default parameters) to search for proteins in the training set that have a sequence similar to *p*'s. The classifier then considers the top ten proteins returned by BLAST (only the top 10 are considered because the text-based classifier is a kNN classifier with *k = *10, and the goal is to provide a fair comparison with the text-based method) and the protein p is assigned to the function class shared by at least three of the training proteins. If there are multiple function classes that fit the criteria, then they are all assigned to the protein that is being classified.

To evaluate prediction performance on proteins that have no associated text, we compiled as our test dataset a set of functionally-annotated proteins that lack associated text in the UniProtKB/Swiss-Prot database. These *textless *proteins (a total of 155 such proteins) are represented using the text features of homologous proteins, as described earlier in the *Methods *section. The *textless *dataset contains *82 *proteins that are annotated with *molecular function *classes and *111 *proteins that are annotated with *biological process *classes. We classify the *textless *proteins using a classifier that was trained over *all *of the 36,536 proteins used in the cross-validation experiments described above.

To the CAFA challenge we have submitted runs performed over the CAFA dataset. The CAFA dataset originally consisted of 48,298 proteins from the UniProtKB/Swiss-Prot database for which no functional annotation was assigned at the time of the CAFA submission deadline (October 15, 2010). Between the submission deadline and the time of assessment (June 2011), several hundreds of the proteins from the CAFA dataset have received experimentally validated functional annotations; these newly annotated proteins were used to evaluate the classifiers.

The final CAFA evaluation dataset thus contains *596 proteins*, of which 436 proteins annotated with at least one *biological process *GO category and 366 proteins annotated with at least one *molecular function *GO category in the UniProtKB/Swiss-Prot database. Notably, 65 of the 596 proteins are "textless" and we use the strategy described before to represent and classify them. As the CAFA results are not provided to the participants protein-by-protein, but rather at the class-level, it is not possible to assess how well we performed specifically on the textless proteins within the CAFA challenge. The performance of our classifier on the CAFA dataset is compared to results provided by the CAFA evaluation from three other classifiers, denoted here as *CAFA-Prior, CAFA-Seq*, and *Gotcha *[6]. *CAFA-Seq *and *GOtcha *are both sequence-based classifiers; they use BLAST to find proteins from UnitProtKB/Swiss-Prot whose sequences are similar to the target's, transferring the functional annotations of the aligned proteins to the target protein. The main difference between the two classifiers is that *CAFA-Seq *uses the *percent identity *between sequences as a confidence score, whereas *GOtcha *bases its score on the sum of negative logs of e-values associated with the alignments between the target protein and the aligned annotated proteins. In contrast, *CAFA-Prior *assigns *every *GO category label to *each *protein in the dataset and uses the prior distribution of the GO categories in UnitProtKB/Swiss-Prot as a confidence score.

A confidence score, associated with the function assigned by the classifier, aims to represent the confidence in the classifier's prediction. Typically, by requiring the classifiers to only report predictions whose confidence scores are above a minimum confidence level, the classifier's precision increases while its recall decreases. Thus, when comparing classifiers, a threshold is set such that, only functions assigned with a confidence score higher than the threshold are evaluated. In the results discussed below, we compare classifiers' performance on the CAFA evaluation dataset using a confidence threshold of 0.95 for *molecular function *classes. For *biological process *classes, we use a lower confidence threshold for *Text-kNN, GOtcha*, and *CAFA-Prior *because no predictions were made at a confidence threshold of 0.95. This issue is discussed in further detail within the Results section.

## Results

### Cross-validation results

The prediction performance of the classifier over individual function classes is measured using the standard metrics of Precision, Recall, and F-measure, as defined below:

$$\begin{array}{c} \begin{array}{c} \mathrm{Recall}=\frac{\mathrm{TP}}{\mathrm{TP}+\mathrm{FN}};\;\mathrm{Precision}=\frac{\mathrm{TP}}{\mathrm{TP}+\mathrm{FP}}; \end{array}\\ F=\frac{2\cdot \mathrm{Precision}\cdot \mathrm{Recall}}{\mathrm{Precision}+\mathrm{Recall}}; \end{array}$$

where *TP, FP*, and *FN *represent the number of true positives, false positives, and false negatives respectively. That is, for a given function class *f, TP *denotes the number of proteins whose function according to UniProtKB/Swiss-Prot is *f *and were labelled as *f *by the classifier; *FP *denotes the number of proteins that were labelled as *f *by the classifier but this label does not match their annotated function in UniProtKB/Swiss-Prot; *FN *denotes to the number of proteins whose function according to UniProtKB/Swiss-Prot is *f *but were mislabelled and assigned to another class by the classifier. We also measure the *overall accuracy *of our classifier, calculated as: *O_acc _= C/n*, where *C *is the number of proteins in the test set that are *correctly *classified and *n *is the total number of proteins in the test set.

The overall prediction accuracy levels of the classifiers *Text-KNN, Base-Prior*, and *Base-Seq *with respect to the *molecular function *classes are 62%, 43%, and 58% while the overall accuracy levels with respect to the *biological process *classes are 17%, 11%, and 28% respectively. The results show that all three classifiers perform much better when classifying *molecular function *than when classifying *biological processes*. The *Text-KNN *classifier outperforms both baseline classifiers for *molecular function *classes and is second to *Base-Seq *for *biological processes*.

The evaluation results, namely the precision, recall, and F-measure, for individual function classes are shown in Tables 3 and 4. Table 3 lists the performance measured for *molecular function *classes, while Table 4 lists performance for *biological process *classes. In both tables, the highest value for each performance measure across the three classifiers is shown in bold. A precision or recall value of 0 associated with a class, indicates that proteins annotated with that class label in UniProtKB/Swiss-Prot are all mislabelled (i.e. assigned another function) by the classifier.

**Table 3:** Prediction performance on *molecular function *classes, over the cross-validation dataset.

Function	# Training Proteins	# Test Proteins	Text-KNN			Base-Prior			Base-Seq		
			
			P	R	F	P	R	F	P	R	F
GO:0005488	10720	2680	0.65	**0.88**	**0.75**	0.63	0.64	0.63	**0.67**	0.75	0.71

GO:0003824	2943	736	**0.52**	0.23	0.32	0.16	0.15	0.15	0.38	**0.29**	**0.33**

GO:0030528	1276	319	0.44	0.24	0.31	0.07	0.07	0.07	**0.49**	**0.37**	**0.42**

GO:0005215	782	196	**0.59**	0.38	**0.46**	0.04	0.04	0.04	0.50	**0.43**	**0.46**

GO:0060089	738	184	**0.39**	0.16	0.22	0.04	0.04	0.04	0.26	**0.27**	**0.27**

GO:0030234	485	121	**0.43**	0.05	0.08	0.03	0.03	0.03	0.16	**0.09**	**0.12**

GO:0005198	334	84	0.04	0.01	0.01	0.02	0.02	0.02	**0.11**	**0.11**	**0.11**

GO:0016247	58	14	**0.60**	**0.24**	**0.35**	0.01	0.01	0.01	0.00	0.00	0.00

GO:0009055	54	14	0.00	0.00	0.00	0.00	0.00	0.00	0.00	0.00	0.00

GO:0045182	21	5	0.00	0.00	0.00	0.00	0.00	0.00	0.00	0.00	0.00

The text-based classifier, *Text-KNN*, is compared with two baselines: *Base-Prior*, and *Base-Seq*. The columns P, R, and F refer, respectively, to the Precision, Recall, and F-measure of the classifier over individual GO categories. A precision and recall values of 0 on a class indicates that all the proteins belonging to that class are misclassified into another class.

**Table 4:** Prediction performance on *biological process classes*, over the cross-validation dataset.

Function	# Training Protein	# Test Protein	Text-KNN			Base-Prior			Base-Seq		
			
			P	R	F	P	R	F	P	R	F
GO:0065007	3626	906	0.23	**0.52**	0.31	0.20	0.24	0.22	**0.32**	0.48	**0.38**

GO:0032502	3338	835	**0.22**	0.19	0.20	0.12	0.17	0.14	**0.22**	**0.24**	**0.23**

GO:0009987	1790	447	0.24	**0.29**	0.26	0.17	0.14	0.15	**0.26**	0.27	**0.27**

GO:0050896	1780	445	**0.25**	**0.16**	**0.19**	0.10	0.10	0.10	0.16	0.09	0.11

GO:0008152	1658	415	0.23	0.14	0.17	0.08	0.06	0.07	**0.28**	**0.34**	**0.31**

GO:0051234	1204	301	0.32	0.20	0.25	0.05	0.05	0.05	**0.44**	**0.45**	**0.45**

GO:0016043	1145	286	0.13	0.05	0.07	0.06	0.05	0.06	**0.15**	**0.12**	**0.13**

GO:0023052	965	241	0.18	0.11	0.14	0.05	0.04	0.04	**0.30**	**0.28**	**0.29**

GO:0032501	606	151	0.12	0.02	0.04	0.04	0.03	0.04	**0.24**	**0.11**	**0.16**

GO:0022414	346	86	**0.51**	**0.15**	**0.24**	0.02	0.02	0.02	0.14	0.03	0.05

GO:0051704	272	68	**0.29**	**0.09**	**0.14**	0.01	0.01	0.01	0.09	0.04	0.05

GO:0040011	170	42	**0.13**	**0.01**	**0.01**	0.01	**0.01**	**0.01**	**1.00**	**0.05**	**0.09**

GO:0040007	165	41	**0.01**	**0.01**	**0.01**	**0.01**	**0.01**	**0.01**	0.00	0.00	0.00

GO:0051179	151	38	**0.03**	**0.01**	**0.01**	**0.01**	**0.01**	**0.01**	0.00	0.00	0.00

GO:0022610	128	32	**0.07**	**0.02**	**0.03**	0.01	0.01	0.01	0.00	0.00	0.00

GO:0008283	118	29	**0.01**	**0.01**	**0.01**	**0.01**	**0.01**	**0.01**	0.00	0.00	0.00

GO:0000003	96	24	0.00	0.00	0.00	**0.01**	**0.01**	**0.01**	0.00	0.00	0.00

GO:0002376	74	19	**0.06**	**0.03**	**0.04**	0.00	0.00	0.00	0.00	0.00	0.00

GO:0016265	64	16	0.00	0.00	0.00	**0.01**	**0.01**	**0.01**	0.00	0.00	0.00

GO:0071554	46	11	**0.38**	**0.08**	**0.13**	0.01	0.00	0.00	0.00	0.00	0.00

GO:0048511	43	11	**0.31**	**0.06**	**0.10**	0.00	0.00	0.00	0.00	0.00	0.00

GO:0023046	35	9	**0.01**	**0.01**	**0.01**	0.00	0.00	0.00	0.00	0.00	0.00

GO:0044085	16	4	**0.01**	**0.01**	**0.01**	0.00	0.00	0.00	0.00	0.00	0.00

GO:0043473	13	3	0.00	0.00	0.00	0.00	0.00	0.00	0.00	0.00	0.00

The text-based classifier, *Text-KNN*, is compared with two baselines: *Base-Prior*, and *Base-Seq*. The columns P, R, and F refer, respectively, to the Precision, Recall, and F-measure of the classifier over individual GO categories. A precision and recall values of 0 on a class indicates that all the proteins belonging to that class are misclassified into another class.

The results shown in Tables 3 and 4 indicate that the text-based classifier outperforms *Base-Prior *(with high statistical significance, see Discussion section) for almost all classes in terms of F-measure, except for the *molecular function *class '*structural molecular activity*'. Out of the 147 test proteins belonging to this class, 141 were misclassified as *'binding*'. The reason for the poor classification performance for this class may also be explained by the fact that 218 out of 418 proteins in this class are annotated as both '*structural molecular activity*' and '*binding*'.

In comparison to the *Base-Seq *classifier, our text-based classifier has a comparable (i.e. no statistically significant difference) - if not higher - precision for most of the *molecular function *classes, but a lower recall and F-measure for all but three classes. Notably, for the three *molecular function *classes that have fewer than 100 associated proteins, *Text-KNN *makes correct predictions for only one out of the three classes, while *Base-Seq *provides no correct predictions at all. For most of the test proteins that belong to these small classes, both classifiers consistently misclassify them as belonging to the majority class, *'binding*'. For the *biological process *classes that have more than 165 associated proteins (12 out of the 24 classes), *Base-Seq *generally performs better in terms of both precision and recall. However, for function classes with fewer than 165 associated proteins, *Base-Seq *does not make any correct predictions because it consistently misclassifies these proteins as belonging to the classes with the largest number of associated proteins. Meanwhile, our text-based classifier is able to correctly predict the function of some of these proteins, albeit with a low average precision of 0.05 and average recall of 0.02.

We also evaluate our classifier's performance on proteins that have no associated abstracts (*textless*). Table 5 shows the prediction performance on textless proteins that are annotated with *molecular function *classes while Table 6 shows the performance on textless proteins that are annotated with *biological process*. Classes that do not contain any textless proteins are not included in this evaluation. As a point of reference, we also show the average performance obtained in the cross-validation study described earlier on these same classes.

**Table 5:** Prediction performance on *molecular function *classes, over the dataset of *textless *proteins.

**Function**	**# Textless Proteins**	**Text-KNN (Textless)**				***Text-KNN (Cross-validation)***		
				
		**P**	**R**	**F**		***P***	***R***	***F***
		
GO:0005488	58	**0.82**	0.47	0.59		*0.65*	***0.88***	***0.75***
		
GO:0003824	9	0.29	**0.56**	**0.38**		***0.52***	*0.23*	*0.32*
		
GO:0030528	1	0.04	**1.00**	0.08		***0.44***	*0.24*	***0.31***
		
GO:0005215	5	0.50	0.20	0.29		***0.59***	***0.38***	***0.46***
		
GO:0060089	7	**0.44**	0.57	**0.50**		*0.39*	*0.16*	*0.22*
		
GO:0005198	2	0.00	0.00	0.00		*0.04*	*0.01*	*0.01*

Prediction performance of *Text-KNN *on proteins that have no associated text is shown in the *Text-KNN (Textless) *column. As a point of reference, the average cross-validation results, denoted as *Text-KNN (Cross-Validation) *as obtained over the whole cross-validation dataset, are shown for comparison only. The columns P, R, and F refer, respectively, to the Precision, Recall, and F-measure of the classifier over individual GO categories. A precision and recall values of 0 on a class indicates that all the proteins belonging to that class are misclassified into another class.

**Table 6:** Prediction performance on *biological process *classes, over the dataset of *textless *proteins.

**Function**	**# Test Proteins**	**Text-KNN (Textless)**				***Text-KNN (Cross-validation)***		
				
		**P**	**R**	**F**		***P***	***R***	***F***
		
GO:0065007	19	**0.28**	**0**.47	**0.35**		*0.23*	***0.52***	*0.31*
		
GO:0032502	18	0.19	**0.22**	**0.21**		***0.22***	*0.19*	*0.20*
		
GO:0009987	8	0.04	0.13	0.06		***0.24***	***0.29***	***0.26***
		
GO:0050896	20	**0.38**	**0.30**	**0.33**		*0.25*	*0.16*	*0.19*
		
GO:0008152	7	**0.29**	**0.29**	**0.29**		*0.23*	*0.14*	*0.17*
		
GO:0051234	9	**0.33**	**0.33**	**0.33**		*0.32*	*0.20*	*0.25*
		
GO:0016043	6	0.00	0.00	0.00		***0.13***	***0.05***	***0.07***
		
GO:0023052	3	0.00	0.00	0.00		***0.18***	***0.11***	***0.14***
		
GO:0032501	9	0.00	0.00	0.00		***0.12***	***0.02***	***0.04***
		
GO:0022414	7	0.00	0.00	0.00		***0.51***	***0.15***	***0.24***
		
GO:0051704	1	0.00	0.00	0.00		*0.00*	*0.00*	*0.00*
		
GO:0040011	3	0.00	0.00	0.00		*0.00*	*0.00*	*0.00*
		
GO:0002376	1	0.00	0.00	0.00		*0.00*	*0.00*	*0.00*

Prediction performance of *Text-KNN *on proteins that have no associated text is shown in the *Text-KNN (Textless) *column. As a point of reference, the average cross-validation results, denoted as *Text-KNN (Cross-Validation) *as obtained over the whole cross-validation dataset, are shown for comparison only. The columns P, R, and F refer, respectively, to the Precision, Recall, and F-measure of the classifier over individual GO categories. A precision and recall values of 0 on a class indicates that all the proteins belonging to that class are misclassified into another class.

For *molecular function *classes, we observe that for the classes 'binding' (GO:0005488) and 'molecular transducer activity' (GO:0060089) the precision on *textless *proteins is better than the average results obtained in the cross validation study, while for the other classes, the precision is only slightly worse. The only exception is the class 'transcription regulator activity', which has only a single textless protein; the precision here is very low (as quite a few 'binding' textless proteins are misclassified into this class) while the recall is 1.00.

For *biological process *proteins, the performance on eight out of the 13 evaluated classes is consistent with that obtained in the cross validation experiments. For the remaining five classes, the precision and recall are both 0.0 on the *textless *dataset. However, we note once again that the classes with 0.0 precision and recall have a relatively small sample of less than 10 *textless *proteins. Nevertheless, despite the small size of the *textless *dataset, the results for the majority of the evaluated classes are consistent with those obtained for proteins that do have associated text. This demonstrates that our classifier can effectively predict function classes for proteins that are *textless*.

### Results from the CAFA runs

The prediction performance of our classifier on the CAFA dataset (which consists of 596 proteins) is presented in Tables 7 and 8. The results for *molecular function *classes are shown in Table 7, while the results for *biological process *classes are shown in Table 8.

**Table 7:** Prediction performance for *molecular function *classes, over the CAFA evaluation dataset. (The number of proteins in each class is shown below each function header)

Function	Text-KNN (confidence = 0.95)			CAFA-Prior (confidence = 0.01)			CAFA-Seq (confidence = 0.95)			GOtcha (confidence = 0.95)		
	
	P	R	S	P	R	S	P	R	S	P	R	S
**binding** (212 proteins)	0.643	0.17	0.87	0.579	**1**	0.00	**0.9**	0.085	**0.987**	0.723	0.16	0.916

**transporter activity** (28 proteins)	0.00	0.00	0.97	0.077	**1**	0.00	0.5	0.036	**0.997**	**0.714**	0.179	0.994

**catalytic activity** (165 proteins)	0.312	0.03	0.95	0.451	**1**	0.00	0.714	0.03	**0.990**	**0.917**	0.067	**0.995**

The text-based classifier, *Text-KNN*, is compared with baseline results provided by the CAFA challenge: *CAFA-Prior, CAFA-Seq*, and *GOtcha*. The confidence threshold used for each classifier is shown under its name in the respective column. A confidence threshold of 0.01 is used for *CAFA-Prior *because the classifier does not make any predictions for the *'transporter activity' *class at higher confidence thresholds.

The columns P, R, and S refer, respectively, to the Precision, Recall, and Specificity of the classifiers over individual classes. Precision and recall values of 0 for a class indicate that all the proteins belonging to that class are misclassified (when the confidence score is 0.95). *CAFA-Prior *always has a specificity value of 0, because it assigns all the proteins to each class, and as such the number of *true negatives *is always 0.

A specificity value that is close to 1, for a class whose precision and recall are both 0, indicates that most proteins in the dataset are not in the class (*true negatives*) and are indeed not assigned to the class. A few proteins from other classes are misclassified into the class (*false positives*), hence the specificity is slightly less than 1.

**Table 8:** Prediction performance for *biological process *classes, over the CAFA evaluation dataset. (The number of proteins in each class is shown below each function header)

Function	Text-KNN (*confidence = 0.75)*			CAFA-Prior (*confidence = 0.01)*			CAFA-Seq (*confidence = 0.95)*			GOtcha (*confidence = 0.14)*		
	
	P	R	S	P	R	S	P	R	S	P	R	S
**biological regulation** (114 proteins)	0.5	0.009	**0.997**	0.261	**1**	0	0**.632**	0.105	0.978	0.404	0.351	0.817

**multi-organism process** (29 proteins)	0.00	0.00	0.939	0.067	**1**	0	0.00	0.00	**0.99**	**0.286**	0.069	0.988

**localization** (60 proteins)	0.2	0.017	**0.989**	0.138	**1**	0	**0.44**	0.067	0.976	0.297	0.317	0.88

**establishment of localization** (38 proteins)	0.25	0.026	**0.992**	0.087	**1**	0	**0.5**	0.105	0.99	0.263	0.395	0.894

**response to stimulus** (106 proteins)	0.125	0.009	**0.979**	0.243	**1**	0	**0.5**	0.047	**0.985**	0.39	0.302	0.848

**developmental process** (83 proteins)	0.00	0.00	**0.997**	**0.19**	**1**	0	**0.556**	0.06	0.989	0.263	0.181	0.881

**multicellular organismal process** (87 proteins)	0.069	0.023	0.923	0.2	**1**	0	**0.625**	0.115	**0.983**	0.343	0.264	0.874

**signalling** (33 proteins)	**0.5**	0.03	**0.998**	0.076	**1**	0	0.25	0.061	0.985	0.077	0.061	0.94

**biological adhesion** (52 proteins)	0.00	0.00	0.971	0.06	**1**	0	0.00	0.00	**0.998**	0.00	0.00	0.993

**cellular component organization** (64 proteins)	0.00	0.00	**0.997**	0.147	**1**	0	**0.286**	0.031	0.987	0.192	0.156	0.887

**cellular process** (368 proteins)	0.857	0.016	**0.985**	0.844	**1**	0	**0.867**	0.071	0.941	0.866	0.829	0.309

**metabolic process** (213 proteins)	0.00	0.00	**0.991**	0.489	**1**	0	0.588	0.047	0.969	**0.633**	0.559	0.691

**reproduction** (25 proteins)	0.083	0.08	0.946	0.057	**1**	0	0.00	0.00	**0.995**	0.214	0.12	0.973

**reproductive process** (25 proteins)	0.083	0.08	0.946	0.057	**1**	0	0.00	0.00	**0.995**	**0.273**	0.12	0.981

The text-based classifier, *Text-KNN*, compared with baseline results provided by the CAFA challenge: *CAFA-Prior, CAFA-Seq*, and *GOtcha*. The confidence threshold used for each classifier is shown under its name in the respective column. The confidence threshold for *Text-kNN, GOtcha*, and *CAFA-Prior *are, respectively, set at 0.75, 0.14, and 0.01 since these classifiers make no predictions for over 75% of the classes at higher confidence thresholds.

The columns P, R, and S refer, respectively, to the Precision, Recall, and Specificity of the classifier over individual classes. Precision and recall values of 0 for a class indicate that all the proteins belonging to that class are misclassified (at the respective confidence level). *CAFA-Prior *always has a specificity value of 0, because it assigns all the proteins to each class, and as such the number of *true negatives *is always 0.

A specificity value that is close to 1, for a class whose precision and recall are both 0, indicates that most proteins in the dataset are not in the class (*true negatives*) and are indeed not assigned to the class. A few proteins from other classes are misclassified into the class (*false positives*), hence the specificity is slightly less than 1.

For *molecular function *classes, the results are shown at a confidence threshold of 0.95 for *Text-kNN, CAFA-Seq and GOtcha*. For *CAFA-Prior*, a confidence threshold of 0.01 is used, because at a confidence threshold of 0.02 *CAFA-Prior *makes no predictions for the *'transporter activity' *class and soon after, at a confidence threshold of 0.14, *CAFA-Prior *makes no predictions for the *'catalytic activity' *class.

For *biological process *classes, only *CAFA-Seq *results are shown at a confidence threshold of 0.95. The results for our classifier, *Text-kNN*, are shown at a threshold of 0.75, while *GOtcha*, and *CAFA-Prior *results are shown at a threshold of 0.14 and 0.01 respectively. These thresholds are chosen because the classifiers make no prediction for over 75% of the classes at a higher confidence level.

Note that because our classifier only assigns proteins to GO categories at the second level of the GO hierarchy, the prediction performance on GO categories that belong to lower (more specific) levels of the GO hierarchy are not shown. Prediction performance is measured by CAFA using *Precision, Recall, and Specificity*. Precision and recall are defined above, whereas specificity is measured as:

$$\mathrm{Specificity}=\frac{\mathrm{TN}}{\mathrm{TN}+\mathrm{FP}}.$$

A specificity value of 1 over a class indicates that all the proteins that are *not annotated *with that class label in UniProtKB/Swiss-Prot are correctly identified as such, and thus there are no false positives.

As shown in Table 7, the precision of our classifier for two of the *molecular function *classes over the CAFA dataset is comparable to the results obtained through cross-validation. Namely, at a confidence score threshold of 0.95, the precision for the '*binding*' class was 0.64 on the cross-validation dataset and 0.74 on the CAFA dataset (which contains 212 'binding' proteins); the precision for the '*catalytic activity*' class was 0.31 on the cross-validation targets and 0.49 on the CAFA targets (which contains 165 'catalaytic activity' proteins). In contrast, for the third class, *'transporter activity' *(28 proteins in the CAFA dataset), the precision shown in Table 6 is 0. However, if we consider predictions made at a lower confidence threshold of 0.8, the precision is 0.24 with a recall of 0.18 compared with a precision of 0.59 and a recall of 0.38 on the cross-validation dataset. Notably, the *'transporter activity' *class is much larger in the cross-validation dataset with a total of 978 proteins as opposed to only 28 proteins in the CAFA dataset. When compared to the baseline classifiers, our text-based classifier has a significantly higher precision than *CAFA-Prior *over the *'binding' *and *'transporter activity' *classes while the *CAFA-Seq *and *GOtcha *classifiers both have a higher precision on all three classes. (Again, we note that we report *CAFA-Prior*'s performance at a very low confidence level, because at a higher confidence threshold it makes no predictions for most classes.)

In terms of recall, *CAFA-Prior *has a recall of 1.0 on all classes at the confidence threshold of 0.01, but its specificity is 0.0 because it assigns *every *GO category label to *each *protein (giving rise to 0 true negatives). *GOtcha *has the highest recall for all three *molecular function *classes when compared to only *CAFA-Seq and Text-kNN*. Our classifier has a slightly higher recall than *CAFA-Seq *on the *'binding' *class but a lower recall on *'catalytic activity' *and '*transporter activity'*.

For the *biological process *classes, as shown in Table 8, *CAFA-Seq *has the highest precision on 11 out of the 14 classes. However, for the '*biological adhesion' *class, neither *CAFA-Seq, GOtcha *nor our classifier made any correct predictions, that is, they all have precision and recall of 0. (Recall that *CAFA-prior *assigns all the proteins into each class, and as such by default always makes some correct predictions at a confidence threshold of 0.01). Moreover, both *CAFA-Seq *and our classifier have precision and recall of 0 for the *'multi-organism process' *class. Even though all three classifiers have a precision and a recall of 0 on these classes, the specificity on those is still very close to 1. This is because the vast majority of proteins belong to other classes and are assigned to other classes, thus keeping true negatives correctly labelled as negatives.

For the *'signalling' *class, our classifier has a significantly (p < 0.05) higher precision than all other three classifiers, while for the *'binding' *class, our classifier has the second highest precision after *CAFA-Seq*. Compared to *CAFA-Prior*, our classifier has a significantly (p < 0.05) higher precision for four of the 14 classes and a slightly higher precision for three of the 14 classes. (We note again though that this comparison is done where the confidence score for our classifier is 0.95 while for *CAFA-Prior *it is only 0.01. When both classifiers are compared at the 0.95 confidence level *CAFA-Prior *makes no predictions, and thus the text-based classifier vacuously outperforms it on all classes). In terms of recall, *GOtcha *once again has the highest recall (second to *CAFA-Prior *which has a recall of 1) while both *CAFA-Seq *and our classifier demonstrate poor recall on all the classes.

## Discussion

The *cross-validation results *above reflect our first attempt at functional classification of proteins while using text as a source of features for representing proteins. These results demonstrate that our classifier performs significantly better (p < 0.05, based on the 2-sample t-test) than a simple baseline classifier, *Base-Prior*, which makes its predictions based solely on the class distribution of the training dataset. Compared to *Base-Seq*, which uses sequence similarity, our text-based classifier has significantly higher precision (p < 0.05), with a lower recall for half of the *molecular function *classes; comparable performance - with no statistically significant difference - for three classes: *'binding' *(GO:0005488), *'electron carrier activity' *(GO:0009055), and *'translation regulator activity' *(GO:0045182); and lower precision and recall for two classes: *'transcription regulator activity' *(GO:0030528), and *'structural molecular activity' *(GO:005198).

With respect to *biological process *classes, our classifier does not perform as well as *Base-Seq *for most classes except for *'reproductive process' *(GO:0022414), *'multi-organism process' *(GO:0051704), *'cell wall organization or biogenesis' *(GO0071554), and *'rhythmic process' *(GO:0048511), where our classifier performs significantly better (p < 0.05).

The results obtained over the textless proteins, demonstrating a level of performance similar for the most part to the one obtained in the cross-validation studies, validates that our strategy for handling textless proteins is indeed effective.

The results provided by the *CAFA evaluation *also show that our text-based classifier *Text-kNN *consistently has a significantly higher precision than the prior-based classifier, *CAFA-Prior*, for *molecular function *classes, and a higher precision for half of the *biological process *classes. However, the sequence-based classifiers, *CAFA-Seq *and *GOtcha*, outperform the text-based classifier on most *molecular function *and *biological process *classes. The exceptions are the *biological process *classes *'biological regulation'*, where *Text-kNN *has a significantly higher precision than the *GOtcha*, classifier; *Text-kNN *also shows a higher precision than both sequence-based classifiers on the *'signalling' *class. Notably, the CAFA results are obtained over a small set of only 596 proteins, while the cross-validation dataset consists of more than 36,000 proteins.

Both the cross-validation and the CAFA evaluations show that our text-based classifier generally has a lower overall precision and recall than a sequence-based classifier. We note that while the classifier based solely on text features does not yield top performance, there are still certain classes on which the text-based classifier shows a higher level of performance than the other classifiers. This suggests that text contains valuable information for identifying particular function classes, and that combining text features with other types of features to make predictions has the potential to lead to improved performance.

As we have noted, the cross-validation results demonstrate poor performance of all three classifiers over most of the *small function classes *(function classes with fewer than 100 proteins). For our text-based classifier, we note that the average number of characteristic terms associated with *small function classes *(average of 121 characteristic terms for both *molecular function *and *biological process *classes) is significantly higher than the average number of distinguishing terms for larger function classes (average of 27 characteristic terms for *molecular function *classes and average of 14 characteristic terms for *biological process *classes). However, the larger set of characteristic terms includes many common, uninformative terms.

For instance, the set of characteristic terms associated with *'electron carrier activity' *(GO:0009055, which has 68 associated proteins, 299 associated abstracts, and 109 characteristic terms) include *human, bovine, chronic, demonstrate*, and *library*. Similarly, *'translation regulatory activity' *(GO:0045182, which has 26 proteins, 110 associated abstracts, and 196 characteristic terms) is associated with *father, male*, and *sperm*. This phenomenon is caused by the low number of proteins - and the correspondingly low number of abstracts - associated with the smaller classes. To illustrate this point, the term *human*, has a probability close to 0.6 to appear in the small number of abstracts associated with the class '*electron carrier activity'*, while having a probability of 0.37 to occur in other classes. Thus, even though the term *human *is a frequent one and is not highly informative, it still appears to be over-represented in the small class, and as such viewed as characteristic to it.

The inclusion of such uninformative terms in the representation, leads to a particularly poor performance in *small function classes*, as the representation of proteins in these classes is dominated by high weights for terms that are often found in abstracts associated with many other classes. The use of the *k-Nearest-Neighbours *classifier in our system, coupled with this representation, leads to misclassifying proteins of small function classes into the larger classes (resulting in low recall), and vice versa (leading to low precision). This is due to high levels of cosine-similarity among protein representations across the different classes, brought about by the abundance of highly popular terms. It is likely that the use of a different classifier rather than k-nearest-neighbours, (for instance, Naïve Bayes or Support Vector Machines, which use a summarizing model to represent each class as opposed to comparison to individual members), will alleviate this problem; this will be done in a follow-up study.

As for the *Base-Seq *classifier, the precision and the recall for the smaller classes are both 0 because the proteins from the *small function classes *are consistently being misclassified into the larger classes based on the BLAST results.

Notably, all three classifiers perform much better on *molecular function *classes than on *biological process *classes. This behaviour is consistently demonstrated using both cross-validation studies and the CAFA evaluation dataset. A possible explanation for the poorer performance on *biological process *classes is that proteins with similar chemical properties and similar molecular functions can often be involved in a broad range of biological processes. For example, protein kinases are involved in *'cellular process' *(GO:0009987), '*metabolic process' *(GO:0008152), *'biological regulation' *(GO:0065007), and *'cellular component organization or biogenesis' *(GO:0071840). Therefore, while both the *Text-KNN *classifier and the *Base-Seq *classifier can rely on the similarity in text features or in sequence (respectively) to identify proteins with similar chemical properties (which often implies similar molecular function), proteins similar in all these respects may still not share the same biological processes. Consequently, the molecular process of the nearest-neighbouring proteins is often not necessarily the one that should be assigned to the newly classified protein.

We note that this issue does not affect the performance of the *molecular function *classifiers, because the molecular function classes are more specific in nature, thus proteins that belong to the same *molecular function *class are often discussed using similar characteristic terminology, and may often have similar sequences,

## Conclusions

We have presented a new system that we have developed toward the CAFA challenge, which is a first attempt to use text features as a basis for classifying proteins into functional categories. This preliminary study demonstrates how a text-based classification system may be used to predict the *molecular function *and *biological processes *of proteins from all organisms. It also utilizes an effective strategy for assigning text to proteins that have no associated text ("textless" proteins), enabling text-based function prediction for such proteins. The current results suggest that text features, and the statistics we employ to select them, can be used successfully when there is a sufficient amount of training data (large classes of proteins, with over 100 proteins per class), and are more suitable for predicting *molecular function *of proteins that for predicting their *biological process*.

The cross-validation results, which use a set of 36,536 proteins, show that on a few of the *molecular function *and *biological process *classes, our system has comparable precision to the sequence-based baseline classifiers. On *molecular function *classes, the results suggest that using text features can yield higher precision and higher overall accuracy. As for *biological process *classes, our classifier does not perform as well as *Base-Seq *on most classes except for *'reproductive process' *(GO:0022414), *'multi-organism process' *(GO:0051704), *'cell wall organization or biogenesis' *(GO:0071554), and *'rhythmic process' *(GO:0048511), where our classifier performs significantly better (p < 0.05).

The CAFA evaluation results (obtained on a set of 596 of proteins) also show that even though our text-based classifier, *Text-KNN *has a lower recall than the sequence-based classifiers, it has significantly higher precision than at least one of the sequence-based classifiers on the classes *'binding', 'signalling' *and *'biological regulation'*.

It is important to note that from the onset, we view text as an important source of available information, but not necessarily as the *best *source of information for classifying proteins. As such, our experiments and their results strongly suggest that integrating text-based classifiers with other classifiers (e.g. sequence- or structure-based ones) is likely to lead to a significant improvement in computational function prediction, in a way similar to that demonstrated for protein subcellular localization [26]. We shall explore such an integrative approach in upcoming work.

The results also suggests that text features are more suitable for predicting the *molecular function *of proteins than the *biological process *of proteins, and that the results are strongly affected by how informative the selected characteristic terms are. An immediate next step in this research (on which we are currently working) is the evaluation of several statistics for selecting effective characteristic terms, in a way that would accommodate accurate classification even for categories with only a small number of associated proteins. Moreover, the procedure through which the k-nearest-neighbour classifier assigns classes to items makes it particularly sensitive to the feature values that represent each item. We expect that some of the problems stemming from the feature selection procedure would be alleviated through the use of model-based classifiers such as the naïve Bayes or Support Vector Machines. This is another direction that we are actively pursuing.

## Competing interests

The authors declare that they have no competing interests.

## Authors' contributions

Conceived and designed the experiments: AW, HS. Performed the experiments: AW. Data Analysis: AW, HS. Manuscript writing: AW, HS.

## Declarations

This article has been published as part of *BMC Bioinformatics *Volume 14 Supplement 3, 2013: Proceedings of Automated Function Prediction SIG 2011 featuring the CAFA Challenge: Critical Assessment of Function Annotations. The full contents of the supplement are available online at http://www.biomedcentral.com/bmcbioinformatics/supplements/14/S3.
